# Exercise intervention for sleep disorders after stroke: A protocol for systematic review and meta-analysis

**DOI:** 10.1097/MD.0000000000025730

**Published:** 2021-04-30

**Authors:** Qin Zhang, Yi Liu, Yin Liang, Dan Yang, Wei Zhang, Liqun Zou, Zhi Wan

**Affiliations:** a Department of Emergency Medicine, Laboratory of Emergency Medicine, Disaster Medical Center; b School of Nursing, West China Hospital, Sichuan University, Chengdu, China.

**Keywords:** exercise, physical activity, sleep disorder, stroke

## Abstract

**Background:**

Sleep disorders after stroke is one of the most common neuropsychiatric complications and is associated with increased risk of death and poor functional outcomes. Some evidence shows that patients with sleep disorders after stroke benefit from exercise intervention. However, this result is still conflicting. This study aims to explore the effects of exercise on sleep disorders after stroke and to establish safe and effective exercise prescriptions.

**Methods and analysis:**

The databases including Google Scholar, Pubmed, Embase, and Cochrane library will be searched using pre-specified search strategies. Randomized controlled trials and non-randomized prospective controlled cohort studies regarding exercise for sleep disorders after stroke will be included. The primary outcome is the Pittsburgh Sleep Quality Index (PSQI) scale. The secondary outcomes are adverse events associated with exercise and all deaths. The methodological quality of each study will be evaluated by the physiotherapy evidence database scale. The heterogeneity will be evaluated using the *I*^2^ test. If *I*^2^ > 50%, random effects models will be used in the analysis; otherwise, fixed effects models will be used to pool the data.

**Results:**

This study will assess the efficacy and safety of exercise for sleep disorders after stroke.

**Conclusions:**

Our findings will be helpful for clinicians to examine the clinical decision-making in the treatment of sleep disorders after stroke using exercise intervention.

**Ethics and dissemination:**

Ethical approval is not required because this study is a secondary analysis. The results of this study will be disseminated through journals and academic exchanges.

**Systematic review registration number:**

INPLASY202130106.

## 1 Introduction

### 1.1 Rationale

Stroke is a common condition that seriously endangers human health, having a high incidence, mortality, and disability rate. It has become the world's second most lethal disease, with 11.8% of global deaths, second only to heart disease, and is also one of the main causes of disability.^[1–4]^ More than half of the cost of unconventional care for stroke patients is a heavy and long-lasting burden on individuals, families, and society.^[5]^ According to global disease burden data, cases of ischemic and hemorrhagic strokes increased by 37% and 47%, respectively, and the incidence of young trends.^[6,7]^

Poststroke sleep disorder (PSSD) is the most common complication of stroke. After a stroke, brain cells are damaged to varying degrees, causing the release of toxic substances that can negatively affect the reticular activation of the brain and damage the sleep-arousal system, leading to sleep disorders. If the patient's sleep quality does not improve over a long time, the patient is highly prone to negative emotions such as anxiety and depression, compounding the psychological and physical suffering, thus damaging motor and sensory functions, and worsening prognosis.^[8]^ Suh et al reported that the incidence of sleep disorders after stroke was as high as 44.20%.^[9]^ Sleep disorders have serious negative effects on the recovery of neurological function and the quality of life of patients with stroke.^[10–13]^ They increase the risk of stroke recurrence,^[14]^ exacerbate neurological deficits, and even increase mortality and cognitive impairment.^[15,16]^ Currently, the treatment of sleep disorders is mainly drug-based, however sleeping pills have side effects, and excessive use can lead to death.^[17]^ Therefore, the use of a safe, effective, economical, and convenient non-drug therapy intervention has become an important issue.

Some studies have found that disturbances in neurotransmitter levels, such as serotonin and norepinephrine, have a certain effect on the pathogenesis of sleep disorders in stroke.^[18,19]^ Exercise controls anxiety and depression,^[20,21]^ increasing the concentration of serotonin in the brain,^[22,23]^ and improving immunity.^[24]^ It has been considered as a supplementary alternative therapy for sleep disorders.^[25]^ Many studies have confirmed that exercise is effective in improving sleep disorders.^[26–29]^ However, due to the different types, frequencies, intensities, times, and places of exercise, there exist differences in the effect of exercise.

### 1.2 Objective

Therefore, the purpose of this systematic review was to evaluate the effectiveness and safety of exercise in improving sleep disorders after a stroke, in order to obtain scientific exercise programs and provide the basis to develop a sports plan.

## 2 Methods

The preferred reporting item for a systematic review and meta-analysis statement will run through our entire study.^[30]^ This study will follow the preferred reporting item for a systematic review and meta-analysis 2015 statement.^[31]^ The program was registered on the international platform of registered systematic review and meta-analysis protocols in April 2021 with registration number: INPLASY202130106 (10.37766/inplasy2021.3.0106), which is accessible at https://inplasy.com. Such systematic reviews and meta-analyses do not require ethical approval, as they do not include any information from participants that infringes on their privacy.

### 2.1 Research criteria included

2.1.1Randomized controlled trials of exercise interventions targeting sleep disorders after stroke will be included in our review. Studies that do not declare being randomized controlled trials but meet the criteria for randomized controlled trials will also be included.

#### 2.1.2 Participant type

According to the World Health Organization diagnostic criteria for stroke,^[32]^ we will include all patients with ischemic and hemorrhagic strokes diagnosed by clinical symptoms and cranial imaging, and assessed by the Pittsburgh Sleep Quality Index (PSQI) scale, with no severe cognitive impairment or mobility disability, regardless of region, race, sex, and different stages of stroke. Repeatedly published literature with poor quality assessment, randomized controlled trials of exercise combined with other therapies, and literature without access to raw data will be excluded.

#### 2.1.3 Interventions

Our systematic review and meta-analysis will be based on the recognition of the application of various forms of exercise interventions in the experimental group, including aerobic exercise, anaerobic exercise, flexion, and extension exercise. Exercise can be single or combined, with no limitations on the time, intensity, and frequency of exercise. The control group should not have received any exercise intervention in the randomized controlled trial.

#### 2.1.4 Main results

The main results of this study are the effects of rehabilitation training for sleep disorders after stroke, principally evaluated by the changes in the scores before and after the PSQI scale,^[33]^ which mainly includes the total sleep quality, subjective sleep quality, sleep time, sleep efficiency, sleep disturbance, sleep drug use, and daytime function.

#### 2.1.5 Secondary outcomes

Adverse events (such as falls, fractures) associated with exercise and all deaths.

### 2.2 Search strategy

#### 2.2.1 Electronic search

The search will be done with the following databases: Google Scholar, Pubmed, Embase, and Cochrane library as data sources. The following search terms will be used:

1. “sleep disorder” OR “'somnipathy” OR “insomnia∗” OR “sleep complaint∗” OR “sleep disturb∗” OR “sleep quality” OR “sleep problem”
2. “stroke” OR “poststroke” OR “poststroke” OR “cerebrovasc∗” OR “brain vasc∗” OR “cerebral vasc∗” OR “apoplexy∗” OR “cva∗”
3. “brain∗” OR “cerebr∗” OR “cerebell” OR “intracerebral” O R “intracranial” OR “subarachnoid”
4. “haemorrhage∗” OR “hemorrhage∗” O R “haematoma∗” OR “hematoma∗” OR “bleed∗”
5. “physical exercise” OR “exercise” OR “physical activity” OR “sport” OR “aerobic” OR “training” OR “exercise training” OR “walking” OR “Tai chi” OR “dancing” OR “Yoga” OR “qigong”.

These search terms will be used alone or in combination, alongside manually searching for relevant studies published from library buildings up to April 2021. The literature retrieval will be conducted independently by 2 researchers and will be searched again before data synthesis.

### 2.3 Data collection and analysis

#### 2.3.1 The study selection

**Figure 1:**
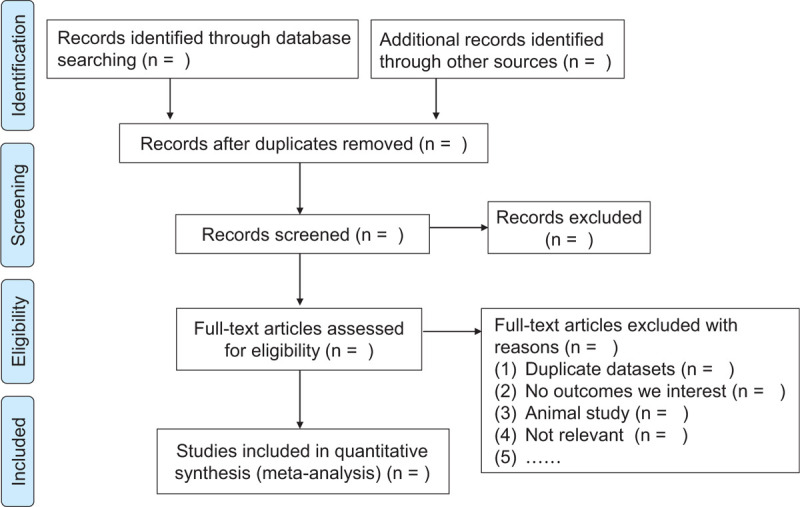
Flow chart of literature screening.

The first 2 researchers will select the topic and abstract independently to determine the standard literature, and then read through the full text to further screen, and record the reasons for excluded literature. When the opinions are inconsistent, the third researcher will decide (Fig. 1).

#### 2.3.2 Data collection

We will develop a unified data extraction table that includes authors, countries, regions, year of publication, sample size, inclusion criteria, patient characteristics (age and sex, body mass index, type of stroke, duration, lesion location, compliance with sleep disorders, and use of sleep disorder drugs), interventions (type, frequency, intensity, and time of exercise), place of intervention (hospital, community, home, and professional institution of exercise), time of follow-up, outcome indicators: score of the PSQI Scale, type and quantity of adverse events, cause, time, and number of deaths. This process will be independently performed by 2 researchers. If the main data are not available, the study will be excluded; the results will be reviewed by a third researcher. If the extraction information is lost or unclear, the author of the literature will be contacted by mail to obtain the data.

#### 2.3.3 Quality assessment

The selected randomized controlled trial will use the Cochrane cooperation bias risk tool to evaluate the quality of the included literature.^[34]^ This tool includes random sequence production, allocation hiding, blind method of subjects and researchers, incomplete outcome data, selective reporting, and other biases. Three grades namely “low bias risk,” “bias uncertainty,” and “high bias risk” will be used to determine each index.

2.3.4 Data analysis will be done using Review Manager 5.3 (Cochrane collaboration) and STATA 16.0 software to calculate means, standard deviations, confidence intervals, and *P* values. For continuous variables, if the results are obtained with the same measuring tool, we will use weighted mean difference analysis; if the same variable uses a different measuring tool, we will use standardized mean difference analysis. For binary variables, the odds ratio (OR) at 95% confidence intervals (CI), will be calculated for all analyses. The bilateral *P* value <.05 will be significant.

#### 2.3.5 Heterogeneity analysis

The heterogeneity among studies is denoted *I*^2^ by *P* values, with *I*^2^ representing the level of heterogeneity between studies. *I*^2^ is negligible in the 0% to 40% range; *I*^2^ is moderate between 40 and 60%; *I*^2^ is high in the 60% to 75% range; and *I*^2^ is extremely high in the 75% to 100% range. If *P* > .1, *I*^2^ < 50%, indicating that there is no heterogeneity in each study, a fixed-effect model is used; if *P* < .1, *I*^2^ ≥ 50, indicating that there is heterogeneity in each study, a random-effect model is adopted; and if the source of heterogeneity cannot be judged and the amount of effect cannot be combined, a descriptive analysis is carried out.

#### 2.3.6 Bias analysis

If the number of randomized controlled trials included is sufficient, the symmetry of the funnel plot will be used to further evaluate the potential publication bias. The examination level is bilateral, and statistical significance is set at *P* < .05.

#### 2.3.7 Group analysis

If the number of studies we include is sufficient, according to the stroke type (hemorrhagic or ischemic), sex (female or male), body mass index (≥25.0 kg/m^2^ or < 25.0 kg/m^2^), places of intervention (hospitals, communities, families and professional institutions for exercise), forms of exercise (aerobic exercise, an anaerobic exercise, flexion and extension exercise or both), follow-up time (1 month, 2–6 months and more than 6 months), and study quality (high or low) for adverse event risk subgroup analysis, the P test of interaction will be used to analyze the difference between subgroups.

#### 2.3.8 Sensitivity analysis

Sensitivity analysis will be used to evaluate the stability and reliability of the meta-analysis and evaluation results. We will observe whether there are significant changes in the synthesis results by excluding each individual randomized controlled trial one at a time. If the change is considered not significant, then the results of the meta-analysis are robust. If the change is considered unstable, vice is true versa.

## 3 Discussion

In recent years, exercise therapy for sleep disorders has become a very safe, effective, economical, and feasible non-drug treatment. More research on the problem of sleep after stroke is needed. The protocol presents a systematic review and meta-analysis approach to review the literature on exercise improvement in PSSD for the first time in a systematic and specific way. The efficacy and safety of exercise in improving PSSD will be evaluated in the meta-analysis, providing valuable information and high-level evidence for improving PSSD. However, its limitations are obvious. We will search only 4 databases, thus omitting some articles published in other databases. The credibility of this systematic review and meta-analysis will be affected by the quality of the included studies. In such studies, missing data is also a common phenomenon that may affect the quality of the study.

## Author contributions

**Conceptualization:** Qin Zhang, Yi Liu, Liqun Zou.

**Funding acquisition:** Liqun Zou.

**Methodology:** Qin Zhang.

**Software:** Yi Liu.

**Supervision:** Wei Zhang, Liqun Zou, Zhi Wan.

**Validation:** Qin Zhang, Yin Liang, Dan Yang, Wei Zhang.

**Writing – original draft:** Qin Zhang, Yi Liu, Yin Liang, Dan Yang, Wei Zhang, Liqun Zou.

**Writing – review & editing:** Qin Zhang, Zhi Wan.
